# MTA Apical Plug and Clinical Application of Anatomic Post and Core for Coronal Restoration: A Case Report

**Published:** 2011-05-15

**Authors:** Rahul Kumar, Suvarna Patil, Upendra Hoshing, Ashish Medha, Rushikesh Mahaparale

**Affiliations:** 1. Department of Conservative Dentistry and Endodontics, Vasantdada Patil Dental College and Hospital, Sangli Maharashtra, India.

**Keywords:** Apexification, Apical Resorption, Fiber Post, MTA, Open Apex, Post Core Technique

## Abstract

Apexification with calcium hydroxide is associated with certain difficulties, such as longer treatment time, risk of tooth fracture and incomplete calcification of apical bridge. Mineral trioxide aggregate (MTA) is an alternative material that can be used for apexification of open apices due to its biocompatibility, non-mutagenicity, non-neurotoxicity, regenerative abilities, and good sealing properties. This case report demonstrates application of MTA apical plug and anatomic post and core for the reconstruction of maxillary central incisor. The patient was recalled after six months and no complications were noted. Periapical radiographs demonstrated good adaptation of anatomical post and core to post space and the complete healing of the periapical lesion. This new technique is particularly advantageous in teeth which have open apex, root canals that are not round, wide canals and thin radicular dentin. It is also useful for who request in patients fewer visits.

## INTRODUCTION

A major problem associated with treating traumatized teeth with necrotic pulps and open apices, is achieving an acceptable seal in the apical area. Apexification has traditionally formed an integral part of the treatment of teeth with necrotic pulps with open apices. Apexification is defined as “a method of inducing a calcified barrier in a root with an open apex or the continued apical development of an incompletely formed root in teeth with necrotic pulp” [1].

Historically, calcium hydroxide was the material of choice used to induce the formation of an apical hard tissue barrier. However, there are several disadvantages of apexification with calcium hydroxide. The treatment requires multiple appointments over an extended period of time which can last from 3-24 months [2], repeated number of dressings necessary to complete closure, outcome may be unpredictable incomplete calcification of bridge, increased risk of fracture after the long-term application of calcium hydroxide, esthetic concerns, and coronal microleakage [3].

Recently, an alternative material mineral trioxide aggregate (MTA) has been introduced. MTA is composed of dicalcium and tricalcium silicate, bismuth oxide, and calcium sulfate. Hydration of the powder results in a fine crystalline gel. This solidifies to a hard structure in approximately 3-4 hours [4]. MTA has several advantages, it can be placed in single visit, is biocompatible, non-mutagenic and non-neurotoxic [5], can induce hard tissue formation [6], and has good sealing properties [7]. Mineral trioxide aggregate (MTA) has become the material of choice in artificial apical barrier procedures [8].

Teeth that have lost substantial amount of crown structure and have wide root canals with weak root dentinal walls are commonly seen in dental practice currently. Prefabricated posts are often used due to their ease of placement and a short clinical application time. But in

such cases a prefabricated post (metal or fiber) will not simulate the root canal anatomy. An improved option may be the application of chair side fabrication of fiber post; its good adaptation, esthetics and retention are advantageous. As a result of it’s precise adaptation to the root canal space, the individualized post is surrounded by thin and uniform layer of resin cement, which creates ideal condition for post retention [9].

In this article we report apexification treatment conducted with MTA apical plug and the clinical application of anatomic post and core for aesthetic rehabilitation of traumatized maxillary right central incisor with a wide root canal.

## CASE REPORT

An 18 year old male patient presented to practice, with the chief complaint of dislodged restoration in maxillary right central incisor. Dental history revealed that the patient had fractured his tooth due to a trauma 5 years ago. At that time the tooth was inappropriately treated and restored, and within one year the restoration was dislodged and the patient had neglected to consult a dentist.

**Figure 1:**
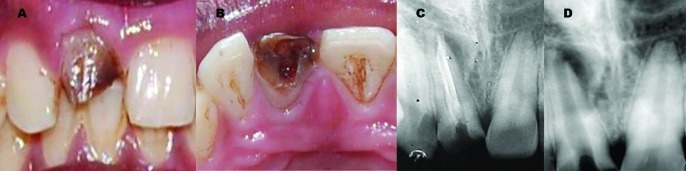
Clinical photographs of the maxillary right central incisor A: Facial view B: Occlusal view. C: Preoperative diagnostic radiograph. D: Radiograph after removal of Gutta-percha

Extraoral examination was unremarkable. On clinical examination, gross destruction of the crown of the maxillary right central incisor was seen and the tooth was tender to percussion (Figure 1A, Figure 1B). Radiographic examination revealed an improperly endodontically treated maxillary right central incisor associated with periapical lesion (Figure 1C). Hence, it was decided to retreat the tooth and to restore it with post and core. Informed consent was obtained from the patient.

Gutta-percha was removed with the help of Hedstrom file (Dentsply/Maillefer, Ballaigues, Switzerland) and radiograph was taken which revealed presence of an open apex with a flared canal (Figure 1D). The patient was concerned about esthetics and wanted to complete his treatment in minimal number of visits, so it was decided to use artificial root-end barrier procedure with MTA and fiber post anatomic post and core for aesthetic rehabilitation of maxillary right central incisor.

**Figure 2:**
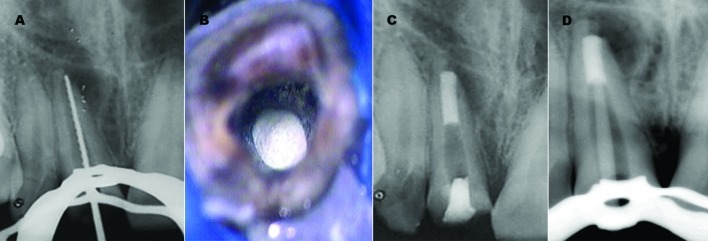
A. Working length radiograph. B: Microscopic view of MTA Plug in root canal. C: Radiographic view showing final MTA plug of 5 mm and temporary coronal seal established with IRM. D: Radiograph showing inadequate adaptation of pre-fabricated fiber post to the post space.

After application of rubber dam (Hygienic Dental Dam, Colténe Whaledent, Germany) and access cavity preparation, working length was determined (Figure 2A). At this stage, size 80 K-file (Dentsply Maillefer, Ballaigues, Switzerland) was loose within the canal and could easily pass beyond the apical limit of the canal. The canal was thoroughly cleaned using 80 K-file (Dentsply Maillefer, Ballaigues, Switzerland) by circumferential filing and 5.25% sodium hypochlorite (Dentpro, Chandigarh, India) irrigation. To obtain canal disinfection prior to MTA placement, canal was dried with paper points (Dentsply Maillefer, Ballaigues, Switzerland), calcium hydroxide (Metapex; Meta Biomed Ltd, Cheongju city, Chungbuk, Korea) was placed, and the access was closed with a sterile cotton pellet followed by a provisional restorative material IRM (Caulk, Dentsply, Milford, DE). The patient was then reappointed for continuation of treatment approximately 1 week from the initial appointment.

At the next appointment, rubber dam was placed as described above. The tooth was re-accessed, and calcium hydroxide was removed with 5.25% NaOCl irrigation (2.5mL) with 27 gauges blunted needle disposable plastic syringe and instrumentation. Before obturation, the canal was irrigated with 5.25% NaOCl, 17% ethylenediaminetetraacetic acid (Glyde File Prep, Densply, France) to remove the smear layer and the canal was then dried with paper points. White ProRoot MTA (Maillfer, Dentsply, Switzerland) was then mixed with distilled water according to manufacturer’s instructions and subsequently placed up to the apex with a fine-tipped MTA carrier and operating microscope (Global Surgical Corporation, St. Louis, MO, USA) (Figure 2B). This procedure was repeated a number of times until the thickness of MTA reached almost 5mm. The plug’s position in canal was checked by radiographs. After application of MTA, a wet paper point was left within the canal for 24 hours, and temporary coronal seal was established with IRM (Caulk, Dentsply, Milford, DE) (Figure 2C).

**Figure 3:**
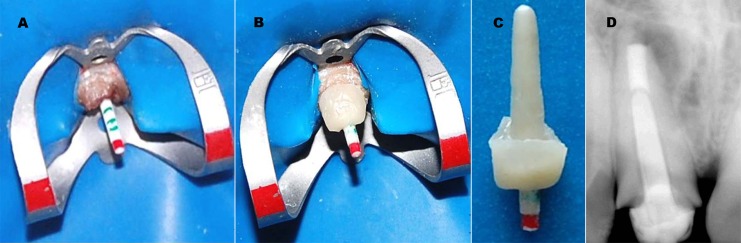
A: Clinical photograph of translucent fiber post with markings for core build-up. B: Procedure showing fabrication of the anatomic post and core. C: Aesthetic anatomic post and core assembly after extraoral curing D: Radiographic view showing fit of anatomic post and core.

At the following appointment, restoration of tooth started with anatomical post and core and metal free ceramic crown. The root canal anatomy of maxillary right central incisor did not permit the use of conventional prefabricated fiber post as the post diameter would not have allowed for good adaptation to the post space, and the resulting thick cement layer would have affected the bond strength (Figure 2D). In addition, the remaining canal dentin thickness was much reduced and cast gold post and core would not have been appropriate as the metal color would have affected the translucency of a subsequent ceramic restoration. Keeping these factors in mind a decision was made to fabricate an aesthetic anatomic post for this tooth. Peeso reamer no. three and four (Dentsply Maillefer, Ballaigues, Switzerland) was used to prepare post space and to remove any undercut that may present on canal walls. The root canal was dried thoroughly and then lubricated with glycerin to act as a separator. A translucent fiber post was selected (Easy Post, Dentsply, Maillefer, France) (Figure 3A) and it was silanized and pretreated with 10% buffered hydrofluoric acid for 2 min followed by the application of silane coupling agent (Ultradent Products Inc, UT, United States), to facilitate the bonding between composite and fiber post material. Resin composite (Filtek™ Z350 3M ESPE, St Paul, MN, USA), was then coated over the post and inserted into the canal adapting it precisely to replicate the canal anatomy. It was light cured intraorally for 5 seconds and subsequently was withdrawn & light cured extraorally for 20 seconds. This procedure continued in increments until the post had a snug fit inside the canal (Figure 3B). Following this, the core was prepared over the post and light curing was performed in order to completely polymerize the composite. Post surface was air-abraded with 50-μm Al2O3 abrasive particles for 10 sec prior to cementation to increase surface roughness and surface area for bonding. After sand-blasting, silane was applied to increasing bond strength (Figure 3C). A radiograph was taken to confirm the marginal fit of the anatomical post and core (Figure 3D).

**Figure 4:**
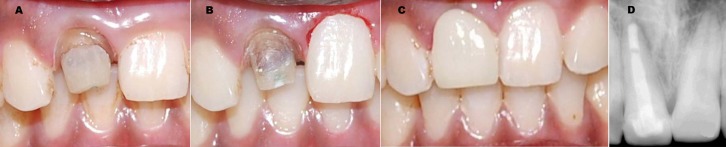
A: Clinical photograph after post cementation. B: Gingivectomy of maxillary left central incisor. C: Clinical photograph after metal-free ceramic crown cementation. D: radiograph after six months showing complete healing of periapical lesion.

The root canal walls were etched with 37% phosphoric acid for 15 seconds and then thoroughly rinsed with water and gently air-dried. The glycerin used as separator was removed at this stage. Bonding agent (Adper Single Bond 2,3M ESPE, St. Paul, USA) was applied into the root canal with a microbrush applicator and cured for 20 seconds. Finally, anatomical post and core cemented with dual cure resin cement (Rely-X ARC, 3M ESPE, St. Paul, USA) (Figure 4A). A final radiograph was taken to confirm the fit of anatomical post and core into the canal. Gingivectomy of maxillary left central incisor was performed to maintain the gingival contour of incisors (Figure 4B).

Temporization was performed and the final metal free ceramic crown was cemented after 1 week (Figure 4C). The patient was recalled after six months and no complications were noted. A periapical radiographs demonstrated that anatomical post and core remained well adapted to post space and the complete healing of periapical lesion (Figure 4D).

## DISCUSSION 

MTA obturation of open-apex teeth overwhelms the defects of conventional apexification procedures. In addition, MTA induces a constant formation of cementum with a high integrity and inclusive periradicular structure. Superiorly, MTA stimulates the periradicular/periodontal tissues’ repair [10][11][12].

The success rate of apexification with calcium hydroxide is approximately 79-96% [13][14]. In human studies, MTA demonstrated healed rates of 81%-100% [15][16][17]. In a retrospective study of open apex teeth obturated with MTA the 1-year success rate was 93.5% and 90.5% for one-visit and two-visit treatments [18].Therefore, MTA has become the material of choice in artificial apical barrier procedures [8].

The present case was suitable for including anatomic post as it had a flared canal and thin radicular dentin. Introducing a conventional fiber post into the canal required either force to round off the canal walls or a thick layer of luting cement to fill up the spaces between the loosely fitting post and the canal walls. This would have subjected the restoration and tooth to adhesive failure and/or debonding of the post [19]. Thus using a post which fit canal shape and a thin uniform layer of cement would increase retention [9].

Esthetic anatomic posts have advantages over the conventional cast metal posts; lower modulus of elasticity protects them from root fracture by reducing the forces that are transferred from the post to the root. A cast metal post would have a wedging effect resulting in root fracture [20]. The aesthetic anatomic post also bonds to the tooth structure forming a monobloc unlike a cast metal post. It can also be made in one visit without any laboratory procedures. The disadvantage of this technique is insufficient clinical data regarding the adhesion and endurance of the restoration.

## CONCLUSION

MTA can be a favorable root filling for apexification. This technique is useful since it is perform in few visits. Also, it results in good periapical seal and allows fabrication of individualized post which provides ample strength and eliminates the laboratory procedures. Further clinical researches are recommended to establish the success of this technique.

## References

[1] Anonymous. (2003). Glossary of endodontic terms. 7th Edition.

[2] Frank AL (1966). Therapy for the divergent pulpless tooth by continued apical formation. J Am Dent Assoc.

[3] Holden DT, Schwartz SA, Kirkpatrick TC, Schindler WG (2008). Clinical outcomes of artificial root-end barriers with mineral trioxide aggregate in teeth with immature apices. J Endod.

[4] Torabinejad M, Hong CU, McDonald F, Pitt Ford TR (1995). Physical and chemical properties of a new root-end filling material. J Endod.

[5] Torabinejad M, Parirokh M (2010). Mineral trioxide aggregate: a comprehensive literature review-part II: leakage and biocompatibility investigations. J Endod.

[6] Apaydin ES, Shabahang S, Torabinejad M (2004). Hard-tissue healing after application of fresh or set MTA as root-end-filling material. J Endod.

[7] Al-Kahtani A, Shostad S, Schifferle R, Bhambhani S (2005). In-vitro evaluation of microleakage of an orthograde apical plug of mineral trioxide aggregate in permanent teeth with simulated immature apices. J Endod.

[8] Shabahang S, Torabinejad M (2000). Treatment of teeth with open apices using mineral trioxide aggregate. Pract Periodontics Aesthet Dent.

[9] Grandini S, Sapio S, Simonetti M (2003). Use of anatomic post and core for reconstructing an endodontically treated tooth: a case report. J Adhes Dent.

[10] Shabahang S, Torabinejad M, Boyne PP, Abedi H, McMillan P (1999). A comparative study of root-end induction using osteogenic protein-1, calcium hydroxide, and mineral trioxide aggregate in dogs. J Endod.

[11] Ham KA, Witherspoon DE, Gutmann JL, Ravindranath S, Gait TC, Opperman LA (2005). Preliminary evaluation of BMP-2 expression and histological characteristics during apexification with calcium hydroxide and mineral trioxide aggregate. J Endod.

[12] Felippe WT, Felippe MC, Rocha MJ (2006). The effect of mineral trioxide aggregate on the apexification and periapical healing of teeth with incomplete root formation. Int Endod J.

[13] Cvek M (1992). Prognosis of luxated non-vital maxillary incisors treated with calcium hydroxide and filled with gutta-percha. A retrospective clinical study. Endod Dent Traumatol.

[14] Kerekes K, Heide S, Jacobsen I (1980). Follow-up examination of endodontic treatment in traumatized juvenile incisors. J Endod.

[15] El-Meligy OA, Avery DR (2006). Comparison of apexification with mineral trioxide aggregate and calcium hydroxide. Pediatr Dent.

[16] Simon S, Rilliard F, Berdal A, Machtou P (2007). The use of mineral trioxide aggregate in one-visit apexification treatment: a prospective study. Int Endod J.

[17] Pace R, Giuliani V, Pini Prato L, Baccetti T, Pagavino G (2007). Apical plug technique using mineral trioxide aggregate: results from a case series. Int Endod J.

[18] Witherspoon DE, Small JC, Regan JD, Nunn M (2008). Retrospective analysis of open apex teeth obturated with mineral trioxide aggregate. J Endod.

[19] Ferrari M, Vichy A, Mannocci F, Mason PN (2000). Retrospective study of clinical behavior of several types of fiber post. Am j Dent.

[20] Stephen Cohen, Kenneth Hargreaves (2006). Pathways of the pulp. 9th Edition.

